# Relevance of comorbidities for main outcomes during different periods of the COVID‐19 pandemic

**DOI:** 10.1111/irv.13240

**Published:** 2024-01-16

**Authors:** José M. Quintana‐Lopez, Lander Rodríguez, Janire Portuondo, Julia García, Maria Jose Legarreta, María Gascón, Nere Larrea, Irantzu Barrio, the COVID‐Health Basque Country Research Group, Janire Portuondo, Julia Garcia, Amaia Bilbao, Idoia Castillo, Susana García‐Gutierrez, Jose M. Quintana, Maria J. Legarreta, María Gascón, Nere Larrea, Cristóbal Esteban, Amaia Aramburu, Pedro Pablo España, Ane Uranga, Dae‐Jin Lee, Lander Rodríguez

**Affiliations:** ^1^ Research Unit, Osakidetza Basque Health Service Galdakao‐Usansolo University Hospital Galdakao Spain; ^2^ Network for Research on Chronicity, Primary Care, and Health Promotion (RICAPPS) Barakaldo Spain; ^3^ Health Service Research Network on Chronic Diseases (REDISSEC) Bilbao Spain; ^4^ Kronikgune Institute for Health Services Research Barakaldo Spain; ^5^ Basque Center for Applied Mathematics, BCAM, Organization and Evaluation Bilbao Spain; ^6^ Osakidetza Basque Health Service Sub‐Directorate for Primary Care Coordination Vitoria‐Gasteiz Spain; ^7^ Biocruces Bizkaia Health Research Institute Barakaldo Spain; ^8^ Basque Government Department of Health Office of Healthcare Planning Vitoria‐Gasteiz Spain; ^9^ Department of Mathematics University of the Basque Country UPV/EHU Leioa Spain

**Keywords:** comorbidity, COVID‐19, healthcare, outcome assessment, patient acuity

## Abstract

**Background:**

Throughout the evolution of the COVID‐19 pandemic, the severity of the disease has varied. The aim of this study was to determine how patients' comorbidities affected and were related to, different outcomes during this time.

**Methods:**

Retrospective cohort study of all patients testing positive for SARS‐CoV‐2 infection between March 1, 2020, and January 9, 2022. We extracted sociodemographic, basal comorbidities, prescribed treatments, COVID‐19 vaccination data, and outcomes such as death and admission to hospital and intensive care unit (ICU) during the different periods of the pandemic. We used logistic regression to quantify the effect of each covariate in each outcome variable and a random forest algorithm to select the most relevant comorbidities.

**Results:**

Predictors of death included having dementia, heart failure, kidney disease, or cancer, while arterial hypertension, diabetes, ischemic heart, cerebrovascular, peripheral vascular diseases, and leukemia were also relevant. Heart failure, dementia, kidney disease, diabetes, and cancer were predictors of adverse evolution (death or ICU admission) with arterial hypertension, ischemic heart, cerebrovascular, peripheral vascular diseases, and leukemia also relevant. Arterial hypertension, heart failure, diabetes, kidney, ischemic heart diseases, and cancer were predictors of hospitalization, while dyslipidemia and respiratory, cerebrovascular, and peripheral vascular diseases were also relevant.

**Conclusions:**

Preexisting comorbidities such as dementia, cardiovascular and renal diseases, and cancers were those most related to adverse outcomes. Of particular note were the discrepancies between predictors of adverse outcomes and predictors of hospitalization and the fact that patients with dementia had a lower probability of being admitted in the first wave.

## 1 INTRODUCTION

The SARS‐CoV‐2 pandemic that began in December 2019^1^ has had unpredictable consequences, posing a threat to public health.^2^, ^3^ From its outset, an attempt was made to understand the pathophysiology of the infection in order to combat the disease.^4^ The numerous and diverse studies performed worldwide required knowledge of data on infections, deaths, pressure on primary care, and hospital bed occupancy.^5^, ^6^, ^7^

Several studies developed models intended to aid in the medical decision‐making process.^8^, ^9^, ^10^, ^11^, ^12^ One of their chief goals was to identify prognostic factors that might help determine which patients were at the highest risk of poor outcomes and to develop care interventions accordingly. Given the changing nature of the infection—due to the different variants of COVID‐19 and the corresponding waves of the disease experienced, knowledge of the similarities and differences between the characteristics of the infection and outcomes between waves remains relatively scarce.^7^, ^13^, ^14^

Several articles have already reported on the role of different comorbidities with regard to different relevant outcomes among COVID‐19 patients. However, this has usually been done in relatively small sample sizes, among hospitalized patients or without exploring differences between the different periods in the pandemic.^15^ In this study, we seek to help understand the characteristics of the COVID‐19 pandemic at different stages, looking at the relationship between various comorbidities and their association with different relevant outcomes.

## 2 METHODS

This is a retrospective study of a cohort of patients diagnosed with COVID‐19 in the Basque Country based on data from the electronic database and health records of Osakidetza, the health service of the Basque Country. This region of Spain has a population of 2.18 million, the vast majority of whom are entitled to healthcare from Osakidetza. The Basque health system is divided into 13 integrated healthcare organizations (IHOs), pooling all primary and hospital care resources in given areas under the same administrative management.

All patients included in this study were resident in the Basque Country and had a SARS‐CoV‐2 infection laboratory‐confirmed by a positive result on the reverse transcriptase‐polymerase chain reaction assay for severe acute respiratory syndrome coronavirus 2 (SARS‐CoV‐2) or a positive antigen test from March 1, 2020, to January 9, 2022. From March 1, 2020, to July 31, 2020, positive IgM or IgG antibody tests performed due to patients having symptoms suggestive of the disease or having had contact with a positive case were also included in the sample. The first positive from each patient was collected, and only patients aged over 18 were included in the study. No patients were excluded. The study protocol was approved by the Ethics Committee of the Basque Country (reference PI2020123). All patient data were kept confidential.

All data on patients under the care of Osakidetza are recorded in a unified electronic database. Analysts retrieved data from all positive cases detected during the study period, including sociodemographic data (age, sex), vaccination dates and doses, baseline comorbidities (all those included in Charlson's comorbidity index,^16^ plus angina, arrhythmia, arterial hypertension, dyslipidemia, asthma, bronchiectasis, cystic fibrosis, interstitial lung disease, lymphoma, leukemia, coagulopathy, inflammatory bowel disease, gastrointestinal bleeding), baseline treatments (based on the Anatomical, Therapeutic, Chemical [ATC] classification system^17^), other background data concerning care provided in hospital or primary care settings, including dates of hospital admission and discharge and whether patients were admitted to an intensive care unit (ICU), and vital status. Comorbidities were identified based on the International Statistical Classification of Diseases and Related Health Problems (ICD) ICD‐9 or 10 codes in patients' records at baseline.^18^ Cancer category was defined as “any malignancy, including lymphoma and leukemia, except malignant neoplasm of skin, and/or metastatic solid tumor.”^19^ Following the launch of the vaccination program, vaccination status was also recorded, as follows: The first dose was considered protective 14 days after inoculation of the vaccine, and the second and third doses were considered the day the inoculation occurred.

Regarding baseline medication, we selected drugs based on ATC codes.^17^ Baseline treatment was defined as any drug prescribed before the patient was diagnosed with SARS‐CoV‐2 infection, with no end date. Data identifying people living in nursing homes were obtained from the Basque Health Department. We also recorded if patients received specific medication for their COVID‐19 (as remdesivir, nirmatrelvir/ritonavir, sotrovimab, or dexamethasone as well).

The outcomes of interest in the study were as follows: (1) hospital admission due to COVID‐19; this was defined as being (a) an admission within 15 days after the patient's testing positive, when the positive test preceded hospitalization, and (b) a positive test in the 21 days following admission, when patients tested positive during hospitalization; (2) death during the 3 months following diagnosis or within a hospital admission; (3) adverse evolution, which included death or ICU admission, within a hospital admission related to a SARS‐CoV‐2 infection diagnosis as defined previously. All patients were followed up until April 9, 2022.

### 2.1 Statistical analysis

Four periods were established from the start of the pandemic until January 9, 2022. The first period included the lockdown period in this country: from March 1 to June 30, 2020. The second period was from July 1, 2020, until December 31, 2020. The third period was from January 1, 2021, to December 13, 2021, the date on which cases of the Omicron variant first began to appear. Finally, the fourth period was from December 14, 2021, until January 9, 2022, during which time the Omicron variant was prevalent.

In order to determine the most relevant variables in each period and study their relationship to the response variables, two different methodologies were applied for each period and outcome. On the one hand, a random forest (RF) algorithm was applied to order the comorbidities based on relevance,^20^ which was measured by means of the distribution of the mean minimal depth,^21^ selecting the 10 comorbidities with the lowest mean minimal depth. This was achieved using the R packages randomForest and randomForestExplainer. On the other hand, logistic regression was used to quantify the effect of each covariate in each outcome variable. Indeed, considering the large sample size, we considered an embedded methodology to develop the final logistic regression model, as follows: (1) Each dataset was randomly divided into k = 10 subsamples, and the process was repeated r = 10 times, so that a total of 100 random subsamples were available, ensuring that there were at least 100 events in each subsample. (2) A logistic regression model was adjusted in each of the 100 subsamples and predictor variables were selected first by a backward AIC stepwise process providing the model with the minimum AIC,^22^ and, second, all those variables rated significant with a 95% confidence level by means of the likelihood ratio test were kept in the model. (3) Those significant variables in at least 25 of the 100 subsamples were considered candidate variables for the final model fitted to the full period dataset sample, and all were significant at alpha = 0.01. (4) The effect of each predictor variable was measured using the odds ratio and each confidence interval in the final model. The discrimination ability of the final logistic regression models in each period was measured by the area under the ROC curve (AUC).^23^ Finally, the effect of different combinations of comorbidities on outcomes was studied through a graphical descriptive analysis of the prevalences in each case. The number of possible combinations of comorbidities is so extensive that only the most relevant ones that presented the outcomes studied were analyzed. All effects were considered significant at *p* < 0.01. All statistical analyses were performed using R© version 4.1.2.

## 3 RESULTS

Online Table 1 provides a description of the sociodemographic and the main clinical characteristics of the whole sample divided by periods. Tables 1, 2, 3 specify the logistic model (LM) predictors accompanied by the order of the relevance of the comorbidities in the RF by period for death, adverse evolution, and hospital admission, respectively. Online Tables 2–4 specify the LM estimates by period for death, adverse evolution, and hospital admission, respectively.

**TABLE 1:** Summary table of predictors of death, and their relevance, by periods of the study.

Variables	First period (*N* = 20,457)	Second period (*N* = 79,941)	Third period (*N* = 140,669)	Omicron period (*N* = 139,014)	All periods (*N* = 380,081)
Gender (M)	X	X	X	X	X
Age (older)	X	X	X	X	X
No COVID‐19 vaccination	NA	NA	X	X	X
Basal comorbidities					
Heart failure	1 X	2 X	1 X	1 X	2 X
Ischemic heart disease	8 X	8	9	7	7
Peripheral vascular disease	6 X	7	7 X	10	9
Cerebrovascular disease	5 X	5	6	9	8
Hemiplegia/paraplegia					
Arterial hypertension	4	4	3	4	4
Dementia	2 X	1 X	2 X	2 X	1 X
Liver disease	10	10		8 X	
Diabetes	7 X	6	5 X	5	6
Kidney disease	3 X	3 X	4 X	3	3
HIV	X				
Cancer	9 X	9 X	8 X	6 X	5 X
Dyslipidemia			10		10
Basal treatments					
Diuretics	X		X	X	
Lipid‐lowering drugs/statins				X—decreased odds	
Heparin				X	
Chronic systemic steroids	X	X	X	X	X
COVID‐19 medication	X	X	X	X	X

*Notes*: X: statistically significant predictor in the logistic multivariable model of Online Table 2. Odds ratios are always >1, except where indicated (decreased odds= > 0 to <1). Numbers indicate order of the relevance of the first 10 comorbidities, as selected by the mean minimal depth of the random forest.

**TABLE 2:** Summary table of predictors of adverse evolution, and their relevance, by periods of the study.

Variables	First period (*N* = 20,457)	Second period (*N* = 79,941)	Third period (*N* = 140,669)	Omicron period (*N* = 139,014)	All periods (*N* = 380,081)
Gender (M)	X	X	X	X	X
Age (older)	X	X	X	X	X
No COVID‐19 vaccination			X	X	X
Basal comorbidities					
Ischemic heart disease	8 X	7	8 X	6	6
Heart failure	1 X	1 X	2 X	1 X	1 X
Peripheral vascular disease	6	8	9	10	8
Cerebrovascular disease	7 X	6	6	8	7
Arterial hypertension	3	4	1	4	4
Dementia	2 X	2 X	3 X	2 X	2 X
Diabetes	5 X	5 X	5 X	5 X	5 X
Kidney disease	4 X	3 X	4 X	3	3
HIV	X				
Liver disease	9			9 X	
Respiratory disease					
Coagulopathy	X				
Cancer	X	9 X	7 X	7 X	9 X
Dyslipidemia	10	10	10		10
Basic treatments					
Diuretics	X			X	
Lipid‐lowering drugs/statins				X decreased odds	
Heparin				X	
Chronic systemic steroids	X	X	X	X	X
COVID‐19 medication	X	X	X	X	X

*Notes*: X: statistically significant predictor in the logistic multivariable model of Online Table 3. Odds ratios are always >1, except where indicated (decreased odds= > 0 to <1). Numbers indicate order of the relevance of the first 10 comorbidities, as selected by the mean minimal depth of the random forest.

**TABLE 3:** Summary table of predictors of hospital admission, and their relevance, by periods of the study.

Variables	First period (*N* = 20,457)	Second period (*N* = 79,941)	Third period (*N* = 140,669)	Omicron period (*N* = 139,014)	All periods (*N* = 380,081)
Gender (M)	X	X	X	X	X
Age (older)	X	X	X	X	X
No COVID‐19 vaccination			X	X	
Basal comorbidities					
Ischemic heart disease	4 X	7 X	6 X	6 X	9 X
Heart failure	8 X	2 X	2 X	1 X	2 X
Cerebrovascular disease		6	9 X	10	7
Hemiplegia/paraplegia	X decreased odds				
Peripheral vascular disease	5	9	8	7	8
Dementia	7 X decreased odds	8	10	5 X	
Arterial hypertension	1 X	1 X	1 X	2 X	1 X
Diabetes	3 X	3 X	3 X	4 X	4 X
Kidney disease	10 X	4 X	4 X	3 X	5 X
Inflammatory bowel disease	X				
Liver disease	9	X	X	X	
Dyslipidemia	2 X	5	5 X		3
Cystic fibrosis		X			
Interstitial lung disease				X	
Respiratory disease	6 X	10	7	8 X	6
Cancer	X	X	X	9 X	10 X
Basic treatments					
RAAS inhibitors	X				
NSAIDs	X	X	X		
Direct oral anticoagulants				X	
Heparin			X	X	
Bronchodilators	X	X	X		
Immunosuppressants	X		X		
Chronic systemic steroids	X	X	X	X	X

*Notes*: X: statistically significant predictor in the logistic multivariable model of Online Table 4. Odds ratios are always >1, except where indicated (decreased odds= > 0 to <1). Numbers indicate order of the relevance of the first 10 comorbidities, as selected by the mean minimal depth of the random forest.

### 3.1 Death

Dementia, heart failure, kidney disease, and cancer were common predictor comorbidities for all periods. In the first period, cerebrovascular, peripheral vascular, and ischemic heart diseases together with diabetes and HIV were also predictors; in the second period liver disease and in the third period diabetes were predictors too; in Omicron diabetes, liver disease and hemiplegia/paraplegia were predictors.

In the RF for all periods, dementia, heart failure, kidney disease, arterial hypertension, cancer, diabetes, ischemic heart disease, cerebrovascular disease, peripheral vascular disease, and dyslipidemia were relevant in that order. In the first, second, and Omicron periods, the only difference is that liver disease was relevant instead of dyslipidemia. However, in the third period, the relevant comorbidities are the same as the ones of all periods. In all periods, heart failure, dementia kidney disease, and arterial hypertension were among the first four in order of relevance.

The comorbidities most frequently related to death were dementia in periods 1 and 2, diabetes in period 3, and cancer in period 4. Cardiovascular disease with diabetes, dementia, or liver or kidney disease was the deadliest combination (Online Figure 1). Generally, the combination of cardiovascular disease, diabetes, or kidney or cerebrovascular diseases with two or three more comorbidities causes higher rates of mortality in periods 1–3, with the number of deaths falling in period 4 (Online Figure 2).

### 3.2 Adverse evolution

Heart failure, dementia, kidney disease, diabetes, and cancer were common predictor comorbidities for all periods. In the first period, peripheral vascular and ischemic heart diseases together with coagulopathy and HIV were also predictors; in the second period, the same common comorbidities were predictors, while in the third, cerebrovascular and ischemic heart diseases were added to the common predictors; in the Omicron period, liver disease was also a predictor.

In the RF for all periods, heart failure, dementia, kidney disease, arterial hypertension, diabetes, ischemic heart disease, cerebrovascular disease, peripheral vascular disease, cancer, and dyslipidemia were relevant in general in that order. In the first period, liver disease was relevant instead of cancer; in the second and third periods, the relevant comorbidities were the same as the ones for all the periods; and in the Omicron period, liver disease was relevant instead of dyslipidemia. In all periods, heart failure, dementia kidney disease, arterial hypertension, and diabetes were among the first five in order of relevance.

The comorbidities most frequently related to adverse evolution were diabetes and dementia in periods 1 and 2, diabetes in period 3, and cancer in period 4, with the worst being the combination of cardiovascular disease with diabetes, dementia, or liver or kidney disease (Online Figure 3). The combination of cardiovascular disease, diabetes, or kidney or cerebrovascular diseases with two or three more comorbidities causes the highest rates of adverse outcomes in periods 1–3, falling in period 4 (Online Figure 4).

### 3.3 Hospital admission

Arterial hypertension, heart failure, diabetes, kidney and ischemic heart diseases, and cancer were common predictor comorbidities for all periods. In the first period, dyslipidemia, respiratory diseases, and inflammatory bowel disease were also predictors, while dementia and hemiplegia/paraplegia were predictors but with decreased odds of hospital admission. When specifically looking at out‐of‐hospital deaths, we found a higher risk for these patients (2.59 (2.29–2.93) and 1.53 (1.2–1.97) OR for dementia and hemiplegia/paraplegia, respectively, in a model adjusted by age, sex, and the Charlson comorbidity index). In the second period, liver disease and cystic fibrosis were also predictors, while in the third, dyslipidemia and cerebrovascular and liver diseases were predictors. In Omicron, dementia (appearing for the first time) and respiratory, liver, and interstitial lung diseases were predictors.

In the RF for all periods, arterial hypertension, heart failure, dyslipidemia, diabetes, kidney, respiratory disease, cerebrovascular disease, peripheral vascular disease, ischemic heart disease, and cancer were relevant in that order. In the first period, dementia and liver disease were relevant instead of cancer and cerebrovascular disease. In the second and third periods, dementia is relevant instead of cancer, while in Omicron, dementia is relevant instead of dyslipidemia. There were more changes in the order of relevance of comorbidities among periods, but arterial hypertension, diabetes, and heart failure were among the first three in the order of relevance with the exception of dyslipidemia in the first period.

The comorbidities most frequently related to hospital admission were diabetes and cancer in all periods, with the worst being the combination of cardiovascular disease with diabetes, dementia, or liver or kidney disease (Online Figure 5). The combination of diabetes or cerebrovascular, cardiovascular, or kidney diseases with two or three more comorbidities caused the highest hospital admission rates in periods 1–3, with the number of admissions falling in period 4 (Online Figure 6).

## 4 DISCUSSION

This large cohort population‐based study of people diagnosed with COVID‐19 outlines several patient profiles based on comorbidities and outcomes that changed slightly over the different periods of the pandemic. Basically, cardiovascular‐related pathologies, renal disease, cancer, and dementia (the latter being the most important) were related to death. The variable profile for adverse evolution was similar, with the addition of diabetes and heart failure (the most important). Finally, the profile for hospital admission was also quite similar, with the addition of a background of respiratory diseases, but (notably) not including dementia.

The detail of comorbidities related to death in patients diagnosed with COVID‐19 infections has already been extensively referred to in other publications.^24^, ^25^ However, to the best of our knowledge, there are few studies of the comorbidities most frequently associated with these outcomes, their most severe combinations, or their importance and role as predictors of such strong outcomes for the different periods of the pandemic.^13^ There were no major differences in the main comorbidities related to death within periods, with the most relevant being dementia^26^ and heart failure^27^ together with kidney disease.^28^ Some other cardiovascular diseases (peripheral vascular disease, cerebrovascular disease, and ischemic heart disease) also played an important role in the first wave,^29^ with HIV featuring only in this period.^30^ The second and third periods had fewer comorbidities related to death, with liver disease and diabetes appearing in all periods,^31^, ^32^ while in the Omicron variant the number of predictive comorbidities increased slightly, with hemiplegia/paraplegia added to the previous predictive variables. The profile for adverse evolution was similar to that previously described, with the addition of diabetes. Based on our data, the first wave of the pandemic appears to have affected a more diverse range of comorbidities —mostly cardiovascular‐related pathologies and dementia. At the beginning of the Omicron wave,^33^ there was an increase in the number of pathologies related to death as compared to the two previous periods. This may be related to the very high and rapid increase in the incidence rate of the infection, although the mortality rate with this variant was much lower due to its lower virulence and also to an effective vaccination program amongst the local population.

In the case of hospital admission, there are several aspects of note. The first is the inverse/decreased odds of dementia and quadriplegia or hemiplegia,^34^ i.e. people with impaired cognitive or physical functioning, with hospital admission in the first wave. This means that patients with these comorbidities were less likely to be admitted to hospital at that time, whereas in the Omicron period, patients with dementia had a higher likelihood of being hospitalized. As almost everywhere, the first wave of the pandemic caused an enormous increase in the pressure on primary care, emergency and hospital services as well as social services, such as nursing homes. This may have resulted in a lower possibility of such patients being admitted. The fact that patients with dementia had a lower chance of being admitted to hospital in the first wave may points to a possible equity problem that should be studied in greater depth.

In general, the main predictors of hospitalization were cardiovascular diseases, as well as arterial hypertension (the most relevant variable), diabetes, renal diseases and cancers. Comparing them to the predictors of death and adverse evolution, we can see that there are more predictors of hospitalization in all periods. Although they share some predictors, it is noticeable that they differ in number and relevance, even in the different periods, from those related to death and adverse evolution. For instance, arterial hypertension did not appear as being predictive nor did it display a high relevance for adverse evolution and death, as it did for hospital admission. This is also the case for respiratory diseases. This may reflect the criteria used by physicians in their clinical decision‐making process at each moment to select the patients to be admitted to hospital, which did not entirely correspond to the comorbidities most related to adverse outcomes. In particular, the special case of dementia should be highlighted: whilst it is one of the most relevant comorbidities related to death and adverse evolution, it did not even appear as a predictor or as an important variable in the RF for hospital admission. The pressure suffered by the Basque health system during the first period necessitated the use of certain decision‐making criteria for hospital admission, which may have affected these patients, as reflected in the inverse likelihood of being hospitalized in the first period of the pandemic. Many of these patients were residents in nursing homes, though this variable was not relevant in the following two periods of the study and was again a predictor and important in the Omicron period for hospital admission, showing what represents a change in the criteria for hospitalizing such patients.

From a statistical point of view, we present our result in two ways: in a more classical fashion, identifying the main predictors of each outcome by using logistic regression models and explaining their effect by means of odds ratios; and understanding the relevance of each comorbidity in the prediction using RF. Using both approaches allows us to consider both the predictability and explainability that different comorbidities have on outcomes.^35^ As presented by others,^36^ the difference in performance between logistic regression and RF is negligible in low dimension (the ratio between number of covariates and sample size is below 0.01) which is the case in our study. Both methods provide quite similar, though not always identical, results. RF did not look for statistical significance but the relevance of each comorbidity. We present the two alongside one another as a complementary way of seeing the role of each comorbidity in each outcome and period of the pandemic.

The strengths of this study include its large sample size —covering almost two years and several variants of the COVID‐19 pandemic—, the selection of three robust and relevant outcomes and the compilation of a large number of data related to sociodemographic variables, comorbidities and prescribed treatments for all people with SARS‐CoV‐2 infection, not restricted to hospitalized patients. Amongst the limitations, we acknowledge the retrospective nature of the study, the lack of some other relevant variables and the limited generalizability of the results. Also, we just included the first positive test to the SARSCOV2 during the study period, though the rate of additional reinfections was low(2.88%).

The results of this study may add clinicians to better identify patients at higher risk of a severe outcome based on their baseline comorbidities. Additionally, the equity problems in access to care of some patients with dementia or hemiplegia/quadriplegia in the first wave of the pandemic should be taken seriously into account in the future to avoid them.

In conclusion, this study provides a full picture of the importance, and combination, of different comorbidities on the rates of death, adverse evolution and hospital admission over four periods of the COVID‐19 pandemic. It highlights the importance of dementia, cardiovascular and renal diseases and cancers as the most relevant comorbidities related to adverse outcomes. However, it also suggests disparities between criteria for hospital admission and adverse outcomes.

## AUTHOR CONTRIBUTIONS

**Jose María Quintana‐Lopez:** Conceptualization (lead); funding acquisition (lead); investigation (equal); methodology (equal); project administration (equal); supervision (lead); writing—original draft (lead); writing—review and editing (lead). **Lander Rodríguez:** Conceptualization (equal); formal analysis (lead); methodology (equal); project administration (equal); writing—original draft (equal); writing—review and editing (equal). **Janire Portuondo‐Jimenez:** Data curation (equal); investigation (equal); project administration (equal); resources (equal); writing—original draft (equal); writing—review and editing (equal). **Julia Garcia‐Asensio:** Data curation (equal); investigation (equal); project administration (equal); resources (equal); writing—original draft (equal); writing—review and editing (equal). **Maria Jose Legarreta:** Data curation (equal); formal analysis (equal); investigation (equal); resources (equal); writing—original draft (equal); writing—review and editing (equal). **María Gascón:** Data curation (equal); formal analysis (equal); investigation (equal); resources (equal); writing—original draft (equal). **Nere Larrea:** Data curation (equal); formal analysis (equal); investigation (equal); resources (equal); writing—original draft (equal); writing—review and editing (equal). **Irantzu Barrio:** Conceptualization (lead); methodology (equal); project administration (equal); resources (equal); supervision (lead); writing—original draft (lead); writing—review and editing (lead). **The COVID‐Health Basque Country Research Group:** Investigation (equal); resources (equal); writing—original draft (equal); writing—review and editing (equal).

## CONFLICT OF INTEREST STATEMENT

The authors declare that they have no conflict of interest.

### PEER REVIEW

The peer review history for this article is available at https://www.webofscience.com/api/gateway/wos/peer-review/10.1111/irv.13240.

## ETHICS STATEMENT

The study protocol was approved by the Ethics Committee of the Basque Country (reference PI2020123). Waive by pandemic situation and retrospective design.

## Supporting information


**Data S1.** Supporting Information

## Data Availability

Data available upon request and agreement with our organization.
