# Supplementary material for: Relevance of comorbidities for main outcomes during different periods of the COVID‐19 pandemic
Source: Influenza Other Respir Viruses. 2024 Jan 16;18(1):e13240. doi: 10.1111/irv.13240 (PMC10790186; doi:10.1111/irv.13240)
Supplement: Supplementary file 1 — Data S1. Supporting Information [file IRV-18-e13240-s001.docx]

*Online Table 1. Descriptive sociodemographic and clinical characteristics of the sample by periods of the COVID-19 pandemic*

| **Variables** | **Period 1 N (%)** | **Period 2 N (%)** | **Period 3 N (%)** | **Omicron (%)** | **P_values 1-2-3** | **P_values 1-2-3-omicron** |
| --- | --- | --- | --- | --- | --- | --- |
| **TOTAL** | **20,457 (5.38%)** | **79,941 (21.03%)** | **140,669 (37.01%)** | **139,014 (36.58%)** |  |  |
| *Sociodemographic variables* |  |  |  |  |  |  |
| **Gender** |  |  |  |  | < 0.0001 | < 0.0001 |
| Female | 12,529 (61.25) | 42,164 (52.74) | 71,344 (50.72) | 73,520 (52.89) |  |  |
| Male | 7,928 (38.75) | 37,777 (47.26) | 69,325 (49.28) | 65,494 (47.11) |  |  |
| **Age (years) categorized** |  |  |  |  | < 0.0001 | < 0.0001 |
| 18-39 | 3,620 (17.70) | 28,149 (35.21) | 56,191 (39.95) | 52,968 (38.10) |  |  |
| 40-49 | 3,423 (16.73) | 15,834 (19.81) | 29,226 (20.78) | 34,951 (25.14) |  |  |
| 50-59 | 4,199 (20.53) | 14,499 (18.14) | 23,440 (16.66) | 27,770 (19.98) |  |  |
| 60-69 | 2,772 (13.55) | 9,142 (11.44) | 15,586 (11.08) | 12,146 (8.74) |  |  |
| 70-79 | 2,256 (11.03) | 5,729 (7.17) | 9,085 (6.46) | 6,353 (4.57) |  |  |
| 80-89 | 2,674 (13.07) | 4,685 (5.86) | 5,250 (3.73) | 3,639 (2.62) |  |  |
| >= 90 | 1,513 (7.40) | 1,903 (2.38) | 1,891 (1.34) | 1,187 (0.85) |  |  |
| **Vaccines** |  |  |  |  | < 0.0001 |  |
| No vaccine |  |  | 95,057 (67.58) | 11,413 (8.21) |  |  |
| 1 dose |  |  | 10,753 (7.64) | 4,583 (3.30) |  |  |
| 2-3 doses |  |  | 34,859 (24.78) | 123,018 (88.49) |  |  |
| *Comorbidities* |  |  |  |  |  |  |
| **Peripheral vascular disease** | 1,058 (5.17) | 2,234 (2.79) | 2,992 (2.13) | 2,363 (1.70) | < 0.0001 | < 0.0001 |
| **Cerebrovascular disease** | 2,484 (12.14) | 4,922 (6.16) | 6,884 (4.89) | 5,809 (4.18) | < 0.0001 | < 0.0001 |
| **Dementia** | 1,685 (8.24) | 2,042 (2.55) | 1,798 (1.28) | 1,104 (0.79) | < 0.0001 | < 0.0001 |
| **Rheumatic disease** | 638 (3.12) | 1,428 (1.79) | 2,114 (1.50) | 1,908 (1.37) | < 0.0001 | < 0.0001 |
| **Peptic ulcer** | 700 (3.42) | 1,590 (1.99) | 2,400 (1.71) | 2,080 (1.50) | < 0.0001 | < 0.0001 |
| **Liver disease** |  |  |  |  | < 0.0001 | < 0.0001 |
| Mild | 1,072 (5.24) | 2,800 (3.50) | 4,359 (3.10) | 3,994 (2.87) |  |  |
| Moderate/Severe | 140 (0.68) | 264 (0.33) | 335 (0.24) | 296 (0.21) |  |  |
| **Diabetes** |  |  |  |  | < 0.0001 | < 0.0001 |
| Yes, without organ damage | 2,151 (10.51) | 4,967 (6.21) | 7,075 (5.03) | 5,312 (3.82) |  |  |
| Yes, with organ damage | 531 (2.60) | 924 (1.16) | 1,266 (0.90) | 853 (0.61) |  |  |
| **Hemiplegia / Paraplegia** | 442 (2.16) | 773 (0.97) | 918 (0.65) | 694 (0.50) | < 0.0001 | < 0.0001 |
| **Kidney** | 2,206 (10.78) | 4,606 (5.76) | 7,086 (5.04) | 6,055 (4.36) | < 0.0001 | < 0.0001 |
| **HIV** | 31 (0.15) | 71 (0.09) | 130 (0.09) | 101 (0.07) | 0.0276 | < 0.01 |
| **Inflammatory bowel disease** | 1,010 (4.94) | 3,237 (4.05) | 5,721 (4.07) | 6,278 (4.52) | < 0.0001 | < 0.0001 |
| **Arterial hypertension** | 7,109 (34.75) | 17,614 (22.03) | 25,611 (18.21) | 21,390 (15.39) | < 0.0001 | < 0.0001 |
| **Dyslipidemia** | 6,494 (31.74) | 17,367 (21.72) | 26,653 (18.95) | 24,842 (17.87) | < 0.0001 | < 0.0001 |
| **Lymphoma** | 940 (4.60) | 4,055 (5.07) | 8,673 (6.17) | 9,712 (6.99) | < 0.0001 | < 0.0001 |
| **Leukemia** | 54 (0.26) | 116 (0.15) | 202 (0.14) | 156 (0.11) | < 0.001 | < 0.0001 |
| **Coagulopathy** | 156 (0.76) | 342 (0.43) | 520 (0.37) | 421 (0.30) | < 0.0001 | < 0.0001 |
| **Gastrointestinal bleeding** | 361 (1.76) | 605 (0.76) | 857 (0.61) | 661 (0.48) | < 0.0001 | < 0.0001 |
| **Asthma** | 2,439 (11.92) | 9,066 (11.34) | 17,175 (12.21) | 17,905 (12.88) | < 0.0001 | < 0.0001 |
| **Cystic fibrosis** | 568 (2.78) | 830 (1.04) | 1,136 (0.81) | 884 (0.64) | < 0.0001 | < 0.0001 |
| **Interstitial lung disease** | 88 (0.43) | 127 (0.16) | 211 (0.15) | 168 (0.12) | < 0.0001 | < 0.0001 |
| **Cancer** | 1,414 (6.91) | 3,432 (4.29) | 5,458 (3.88) | 4,928 (3.54) | < 0.0001 | < 0.0001 |
| **Respiratory disease** | 3,693 (18.05) | 12,207 (15.27) | 22,251 (15.82) | 22,512 (16.19) | < 0.0001 | < 0.0001 |
| **Ischemic heart disease** | 1,423 (6.96) | 2,736 (3.42) | 3,944 (2.80) | 3,129 (2.25) | < 0.0001 | < 0.0001 |
| **Heart failure** | 1,819 (8.89) | 2,992 (3.74) | 3,964 (2.82) | 2,793 (2.01) | < 0.0001 | < 0.0001 |
| *Basic treatments* |  |  |  |  |  |  |
| **Cardiovascular** | 986 (4.82) | 2,045 (2.56) | 3,152 (2.24) | 2,846 (2.05) | < 0.0001 | < 0.0001 |
| **Antihypertensive** | 249 (1.22) | 507 (0.63) | 816 (0.58) | 564 (0.41) | < 0.0001 | < 0.0001 |
| **Diuretics** | 2,352 (11.50) | 4,098 (5.13) | 5,105 (3.63) | 3,421 (2.46) | < 0.0001 | < 0.0001 |
| **Beta-blockers** | 1,755 (8.58) | 3,821 (4.78) | 5,679 (4.04) | 4,615 (3.32) | < 0.0001 | < 0.0001 |
| **Calcium channel blockers** | 1,154 (5.64) | 2,725 (3.41) | 3,791 (2.69) | 2,850 (2.05) | < 0.0001 | < 0.0001 |
| **System inhibitors** | 4,302 (21.03) | 11,337 (14.18) | 16,771 (11.92) | 13,879 (9.98) | < 0.0001 | < 0.0001 |
| **Lipid lowering drugs/statins** | 3,609 (17.64) | 9,738 (12.18) | 14,333 (10.19) | 12,346 (8.88) | < 0.0001 | < 0.0001 |
| **NSAIDs** | 3,605 (17.62) | 12,654 (15.83) | 23,276 (16.55) | 25,161 (18.10) | < 0.0001 | < 0.0001 |
| **Direct oral anticoagulants** | 3,903 (19.08) | 8,200 (10.26) | 11,745 (8.35) | 8,469 (6.09) | < 0.0001 | < 0.0001 |
| **Antiplatelets** | 2,225 (10.88) | 4,406 (5.51) | 6,200 (4.41) | 4,995 (3.59) | < 0.0001 | < 0.0001 |
| **Heparin** | 330 (1.61) | 1,343 (1.68) | 2,319 (1.65) | 1,234 (0.89) | 0.755 | < 0.0001 |
| **Broncodilators** | 2,235 (10.93) | 6,996 (8.75) | 12,149 (8.64) | 12,690 (9.13) | < 0.0001 | < 0.0001 |
| **Immunosuppressants** | 280 (1.37) | 712 (0.89) | 1,207 (0.86) | 1,195 (0.86) | < 0.0001 | < 0.0001 |
| **Chronic systemic steroids** | 939 (4.59) | 1,543 (1.93) | 2,621 (1.86) | 2,450 (1.76) | < 0.0001 | < 0.0001 |
| **COVID-19 Medication** | 2,743 (13.41) | 3,224 (4.03) | 6,886 (4.90) | 1124 (0.81) | < 0.0001 | < 0.0001 |
| *Output variables* |  |  |  |  |  |  |
| **Hospitalization** | 5,486 (26.82) | 6,943 (8.69) | 10,951 (7.78) | 2,236 (1.61) | < 0.0001 | < 0.0001 |
| **Covid death** | 1,678 (8.20) | 1,764 (2.21) | 1,901 (1.35) | 584 (0.42) | < 0.0001 | < 0.0001 |
| **Bad progress** | 2,028 (9.91) | 2,334 (2.92) | 3,112 (2.21) | 761 (0.55) | < 0.0001 | < 0.0001 |

First period from March 1 to June 30, 2020. Second period from July 1st until December 28, 2020. Third period from December 29, 2020 to December 13, 2021. Fourth period (Omicron) from December 14, 2021 to January 9, 2022.

COVID-19 medication: remdesivir, nirmatrelvir/ritonavir, sotrovimab or dexamethasone.

*Online Table 2. Multivariable logistic regression analysis of predictors of death by periods of the study.*

| **Variables** | **First period** | | **Second period** | | **Third period** | | **Omicron** | |
| --- | --- | --- | --- | --- | --- | --- | --- | --- |
|  | **OR (99% CI)** | **p** | **OR (99% CI)** | **p** | **OR (99% CI)** | **p** | **OR (99% CI)** | **p** |
| *Sociodemographic variables* |  |  |  |  |  |  |  |  |
| **Gender** Male (vs female) | 1.81 (1.54-2.14) | <0.0001 | 1.9 (1.64-2.21) | <0.0001 | 1.62 (1.4-1.87) | <0.0001 | 1.74 (1.35-2.24) | <0.0001 |
| **Age (years) categorized** |  |  |  |  |  |  |  |  |
| 18-39 | Ref | - | Ref | - | Ref | - | Ref | - |
| 40-49 | 1.44 (0.42-4.96) | 0.4514 | 6.48 (1.98-21.21) | <0.0001 | 6.37 (2.09-19.43) | <0.0001 | 3.05 (0.8-11.58) | 0.0309 |
| 50-59 | 3.05 (1.05-8.87) | 0.0069 | 15.49 (5.21-46.05) | <0.0001 | 24.23 (8.9-65.99) | <0.0001 | 10.38 (3.17-33.97) | <0.0001 |
| 60-69 | 13.23 (4.88-35.85) | <0.0001 | 55.55 (19.39-159.18) | <0.0001 | 74.88 (28.02-200.13) | <0.0001 | 35.85 (11.31-113.65) | <0.0001 |
| 70-79 | 38.55 (14.45-102.83) | <0.0001 | 142.56 (50.3-404) | <0.0001 | 193.54 (72.79-514.63) | <0.0001 | 92.57 (29.45-291.03) | <0.0001 |
| 80-89 | 76.8 (28.9-204.11) | <0.0001 | 413.87 (146.84-1166.56) | <0.0001 | 499.56 (187.51-1330.95) | <0.0001 | 197.41 (63.14-617.17) | <0.0001 |
| >= 90 | 123.07 (46.07-328.73) | <0.0001 | 891.44 (314.98-2522.92) | <0.0001 | 1264 (471.59-3387.89) | <0.0001 | 582.88 (184.4-1842.42) | <0.0001 |
| **Vaccines** |  |  |  |  |  |  |  |  |
| 2-3 doses |  |  |  | - | Ref | - | Ref | - |
| 1 dose |  |  |  |  | 1.42 (1.09-1.85) | 7e-04 | 1.93 (0.87-4.25) | 0.0328 |
| No dose |  |  |  |  | 1.89 (1.62-2.22) | <0.0001 | 3.04 (2.12-4.35) | <0.0001 |
| *Basal comorbidities* |  |  |  |  |  |  |  |  |
| **Peripheral vascular disease** | 1.45 (1.16-1.82) | <0.0001 |  |  | 1.52 (1.25-1.86) | <0.0001 |  |  |
| **Cerebrovascular disease** | 1.3 (1.1-1.54) | <0.0001 |  |  |  |  |  |  |
| **Dementia** | 2.05 (1.71-2.46) | <0.0001 | 2.81 (2.35-3.35) | <0.0001 | 2.52 (2.07-3.07) | <0.0001 | 2.61 (1.85-3.69) | <0.0001 |
| **Liver disease** |  |  |  |  |  |  |  |  |
| No |  |  |  |  |  | - | Ref | - |
| Mild |  |  |  |  |  |  | 1.85 (1.25-2.72) | <0.0001 |
| Moderate/Severe |  |  |  |  |  |  | 4.6 (2.3-9.17) | <0.0001 |
| **Diabetes** |  |  |  |  |  |  |  |  |
| No | Ref | - |  |  | Ref | - |  |  |
| Yes-without organ damage | 1.17 (0.97-1.4) | 0.0278 |  |  | 1.34 (1.14-1.59) | <0.0001 |  |  |
| Yes-with organ damage | 1.43 (1.05-1.93) | 0.0025 |  |  | 1.73 (1.31-2.27) | <0.0001 |  |  |
| **Hemiplegia / Paraplegia** |  |  |  |  |  |  |  |  |
| **Kidney** | 1.33 (1.11-1.59) | <0.0001 | 1.71 (1.45-2.01) | <0.0001 | 1.52 (1.29-1.8) | <0.0001 |  |  |
| **HIV** | 23.94 (6.72-85.27) | <0.0001 |  |  |  |  |  |  |
| **Cancer** | 1.7 (1.36-2.13) | <0.0001 | 2.17 (1.78-2.64) | <0.0001 | 2.26 (1.88-2.71) | <0.0001 | 2.75 (2.03-3.73) | <0.0001 |
| **Ischemic heart disease** | 1.31 (1.07-1.6) | 7e-04 |  |  |  |  |  |  |
| **Heart failure** | 1.33 (1.09-1.62) | 2e-04 | 1.92 (1.62-2.27) | <0.0001 | 1.68 (1.4-2.02) | <0.0001 | 1.89 (1.36-2.63) | <0.0001 |
| *Basal treatments* |  |  |  |  |  |  |  |  |
| **Diuretics** | 1.27 (1.06-1.53) | 7e-04 |  |  | 1.28 (1.08-1.53) | 3e-04 | 1.96 (1.43-2.68) | <0.0001 |
| **Lipid lowering drugs/statins** |  |  |  |  |  |  | 0.66 (0.5-0.87) | 1e-04 |
| **Heparin** |  |  |  |  |  |  | 2.51 (1.48-4.25) | <0.0001 |
| **Chronic systemic steroids** | 1.69 (1.32-2.16) | <0.0001 | 2.78 (2.17-3.57) | <0.0001 | 4.01 (3.23-4.99) | <0.0001 | 4.24 (2.99-6.01) | <0.0001 |
| **COVID-19 Medication** | 1.89 (1.54-2.32) | <0.0001 | 4.37 (3.67-5.21) | <0.0001 | 5.07 (4.37-5.88) | <0.0001 | 13.11 (9.68-17.76) | <0.0001 |
| **AUC** | 0.8997 (0.8922-0.9071) | | 0.9543 (0.9497-0.9589) | | 0.965 (0.9613-0.9687) | | 0.9678 (0.958-0.9776) | |

First period from March 1 to June 30, 2020. Second period from July 1st until December 28, 2020. Third period from December 29, 2020 to December 13, 2021. Fourth period (Omicron) from December 14, 2021 to January 9, 2022.

COVID-19 medication: remdesivir, nirmatrelvir/ritonavir, sotrovimab or dexamethasone.

*Online Table 3. Multivariable logistic regression analysis of predictors of adverse evolution by periods of the study.*

| **Variables** | **First period** | | **Second period** | | **Third period** | | **Omicron** | |
| --- | --- | --- | --- | --- | --- | --- | --- | --- |
|  | **OR (99% CI)** | **p** | **OR (99% CI)** | **p** | **OR (99% CI)** | **p** | **OR (99% CI)** | **p** |
| *Sociodemographic variables* |  |  |  |  |  |  |  |  |
| **Gender** |  |  |  |  |  |  |  |  |
| Male (vs female) | 1.94 (1.67-2.24) | <0.0001 | 1.96 (1.72-2.23) | <0.0001 | 1.68 (1.5-1.88) | <0.0001 | 1.7 (1.35-2.14) | <0.0001 |
| **Age (years) categorized** |  |  |  |  |  |  |  |  |
| 18-39 | Ref | - | Ref | - | Ref | - | Ref | - |
| 40-49 | 1.6 (0.84-3.03) | 0.0588 | 2.82 (1.76-4.51) | <0.0001 | 2.39 (1.78-3.2) | <0.0001 | 3.09 (1.52-6.27) | <0.0001 |
| 50-59 | 2.6 (1.46-4.61) | <0.0001 | 5.42 (3.52-8.33) | <0.0001 | 4.63 (3.55-6.02) | <0.0001 | 5.68 (2.91-11.09) | <0.0001 |
| 60-69 | 6.67 (3.86-11.52) | <0.0001 | 11.28 (7.44-17.1) | <0.0001 | 8.23 (6.36-10.65) | <0.0001 | 14.06 (7.29-27.1) | <0.0001 |
| 70-79 | 13.07 (7.63-22.41) | <0.0001 | 17.49 (11.53-26.51) | <0.0001 | 12.77 (9.85-16.57) | <0.0001 | 26.17 (13.46-50.88) | <0.0001 |
| 80-89 | 21.34 (12.47-36.53) | <0.0001 | 37.01 (24.51-55.9) | <0.0001 | 23.07 (17.62-30.19) | <0.0001 | 45.26 (23.28-88.01) | <0.0001 |
| >= 90 | 35.89 (20.77-62.01) | <0.0001 | 88.99 (58.22-136.01) | <0.0001 | 68.9 (51.28-92.58) | <0.0001 | 148.66 (75.01-294.6) | <0.0001 |
| **Vaccines** |  |  |  |  |  |  |  |  |
| 2-3 doses |  |  |  |  | Ref | - | Ref | - |
| 1 dose |  |  |  |  | 1.5 (1.2-1.89) | <0.0001 | 1.78 (0.91-3.5) | 0.0273 |
| No dose |  |  |  |  | 2.32 (2.02-2.67) | <0.0001 | 4.29 (3.21-5.72) | <0.0001 |
| *Comorbidities* |  |  |  |  |  |  |  |  |
| **Peripheral vascular disease** |  |  |  |  |  |  |  |  |
| **Cerebrovascular disease** | 1.28 (1.09-1.5) | <0.0001 |  |  |  |  |  |  |
| **Dementia** | 1.98 (1.66-2.37) | <0.0001 | 2.72 (2.28-3.25) | <0.0001 | 2.39 (1.96-2.93) | <0.0001 | 2.57 (1.8-3.68) | <0.0001 |
| **Liver disease** |  |  |  |  |  |  |  |  |
| No | Ref | - | Ref | - | Ref | - | Ref | - |
| Mild |  |  |  |  |  |  | 1.42 (0.97-2.09) | 0.0189 |
| Moderate/Severe |  |  |  |  |  |  | 3.74 (1.88-7.44) | <0.0001 |
| **Diabetes** |  |  |  |  |  |  |  |  |
| No | Ref | - | Ref | - | Ref | - | Ref | - |
| Yes, without organ damage | 1.27 (1.07-1.5) | 3e-04 | 1.3 (1.11-1.52) | <0.0001 | 1.44 (1.25-1.66) | <0.0001 | 1.76 (1.31-2.36) | <0.0001 |
| Yes, with organ damage | 1.42 (1.07-1.9) | 0.0017 | 1.88 (1.43-2.47) | <0.0001 | 1.6 (1.24-2.08) | <0.0001 | 2.17 (1.3-3.63) | <0.0001 |
| **Kidney** | 1.32 (1.12-1.57) | <0.0001 | 1.59 (1.36-1.86) | <0.0001 | 1.47 (1.27-1.71) | <0.0001 |  |  |
| **HIV** | 8.36 (2.7-25.83) | <0.0001 |  |  |  |  |  |  |
| **Coagulopathy** | 2.35 (1.38-4) | <0.0001 |  |  |  |  |  |  |
| **Cancer** | 1.54 (1.25-1.9) | <0.0001 | 1.81 (1.5-2.18) | <0.0001 | 1.95 (1.66-2.31) | <0.0001 | 2.39 (1.77-3.24) | <0.0001 |
| **Ischemic heart disease** | 1.48 (1.22-1.79) | <0.0001 |  |  | 1.46 (1.23-1.73) | <0.0001 |  |  |
| **Heart failure** | 1.31 (1.08-1.58) | 2e-04 | 1.72 (1.45-2.03) | <0.0001 | 1.6 (1.36-1.88) | <0.0001 | 1.88 (1.36-2.61) | <0.0001 |
| *Basic treatments* |  |  |  |  |  |  |  |  |
| **Diuretics** | 1.31 (1.08-1.58) | 2e-04 |  |  |  |  | 1.84 (1.35-2.51) | <0.0001 |
| **Lipid lowering drugs/statins** |  |  |  |  |  |  | 0.61 (0.46-0.8) | <0.0001 |
| **Heparin** |  |  |  |  |  |  | 2.4 (1.44-4) | <0.0001 |
| **Chronic systemic steroids** | 1.6 (1.27-2.02) | <0.0001 | 2.42 (1.91-3.06) | <0.0001 | 3.05 (2.5-3.72) | <0.0001 | 3.51 (2.49-4.96) | <0.0001 |
| **COVID-19 Medication** | 2.92 (2.45-3.47) | <0.0001 | 8.69 (7.51-10.05) | <0.0001 | 12.63 (11.27-14.16) | <0.0001 | 28.47 (21.92-36.98) | <0.0001 |
| **AUC** | 0.8674 (0.8586-0.8761) | | 0.9314 (0.9253-0.9375) | | 0.9437 (0.9392-0.9483) | | 0.9605 (0.9499-0.9712) | |

First period from March 1 to June 30, 2020. Second period from July 1st until December 28, 2020. Third period from December 29, 2020 to December 13, 2021. Fourth period (Omicron) from December 14, 2021 to January 9, 2022.

COVID-19 medication: remdesivir, nirmatrelvir/ritonavir, sotrovimab or dexamethasone.

*Online Table 4. Multivariable logistic regression analysis of predictors of hospitalization by periods of the study.*

| **Variables** | **First period** | | **Second period** | | **Third period** | | **Omicron** | |
| --- | --- | --- | --- | --- | --- | --- | --- | --- |
|  | **OR (99% CI)** | **p** | **OR (99% CI)** | **p** | **OR (99% CI)** | **p** | **OR (99% CI)** | **p** |
| *Sociodemographic variables* |  |  |  |  |  |  |  |  |
| **Gender** |  |  |  |  |  |  |  |  |
| Male (vs female) | 2.33 (2.12-2.56) | <0.0001 | 1.66 (1.55-1.79) | <0.0001 | 1.58 (1.49-1.67) | <0.0001 | 1.43 (1.27-1.62) | <0.0001 |
| **Age (years) categorized** |  |  |  |  |  |  |  |  |
| 18-39 | Ref | - | Ref | - | Ref | - | Ref | - |
| 40-49 | 1.78 (1.45-2.18) | <0.0001 | 2.1 (1.8-2.45) | <0.0001 | 2.55 (2.3-2.84) | <0.0001 | 2.21 (1.75-2.8) | <0.0001 |
| 50-59 | 2.32 (1.92-2.81) | <0.0001 | 3.52 (3.05-4.06) | <0.0001 | 4.25 (3.84-4.7) | <0.0001 | 3.33 (2.65-4.19) | <0.0001 |
| 60-69 | 4.66 (3.82-5.69) | <0.0001 | 6.14 (5.31-7.1) | <0.0001 | 6.85 (6.15-7.63) | <0.0001 | 7.54 (5.97-9.52) | <0.0001 |
| 70-79 | 7.57 (6.12-9.36) | <0.0001 | 9.79 (8.4-11.41) | <0.0001 | 10.68 (9.5-12.01) | <0.0001 | 10.5 (8.12-13.58) | <0.0001 |
| 80-89 | 5.33 (4.27-6.65) | <0.0001 | 14.6 (12.45-17.12) | <0.0001 | 16.89 (14.81-19.26) | <0.0001 | 15.74 (11.97-20.71) | <0.0001 |
| >= 90 | 3.1 (2.41-3.99) | <0.0001 | 13.14 (10.82-15.95) | <0.0001 | 19.34 (16.23-23.04) | <0.0001 | 22.57 (16.18-31.49) | <0.0001 |
| **Vaccines** |  |  |  |  |  |  |  |  |
| 2-3 doses |  |  |  |  | Ref | - | Ref | - |
| 1 dose |  |  |  |  | 1.49 (1.31-1.69) | <0.0001 | 3.5 (2.61-4.71) | <0.0001 |
| No dose |  |  |  |  | 3.61 (3.34-3.9) | <0.0001 | 10.05 (8.69-11.62) | <0.0001 |
| *Comorbidities* |  |  |  |  |  |  |  |  |
| **Cerebrovascular disease** |  |  |  |  | 1.25 (1.13-1.37) | <0.0001 |  |  |
| **Dementia** | 0.58 (0.49-0.69) | <0.0001 |  |  |  |  | 1.62 (1.23-2.12) | <0.0001 |
| **Liver disease** |  |  |  |  |  |  |  |  |
| No |  |  | Ref | - | Ref | - | Ref | - |
| Mild |  |  | 1.44 (1.25-1.65) | <0.0001 | 1.3 (1.16-1.46) | <0.0001 | 1.42 (1.14-1.77) | <0.0001 |
| Moderate/Severe |  |  | 1.86 (1.29-2.69) | <0.0001 | 2.82 (2.04-3.88) | <0.0001 | 2.07 (1.23-3.49) | 3e-04 |
| **Diabetes** |  |  |  |  |  |  |  |  |
| No | Ref | - | Ref | - | Ref | - | Ref | - |
| Yes, without organ damage | 1.27 (1.1-1.46) | <0.0001 | 1.45 (1.31-1.61) | <0.0001 | 1.46 (1.33-1.6) | <0.0001 | 1.38 (1.15-1.65) | <0.0001 |
| Yes, with organ damage | 1.32 (1.01-1.72) | 0.0072 | 1.97 (1.61-2.41) | <0.0001 | 1.89 (1.58-2.26) | <0.0001 | 1.64 (1.19-2.27) | <0.0001 |
| **Hemiplegia / Paraplegia** | 0.61 (0.46-0.82) | <0.0001 |  |  |  |  |  |  |
| **Kidney** | 1.25 (1.08-1.45) | <0.0001 | 1.52 (1.36-1.7) | <0.0001 | 1.4 (1.27-1.55) | <0.0001 | 1.65 (1.38-1.98) | <0.0001 |
| **Inflammatory bowel disease** | 1.4 (1.15-1.71) | <0.0001 |  |  |  |  |  |  |
| **Arterial hypertension** | 1.29 (1.13-1.47) | <0.0001 | 1.22 (1.12-1.33) | <0.0001 | 1.2 (1.12-1.29) | <0.0001 | 1.37 (1.18-1.6) | <0.0001 |
| **Dyslipidemia** | 1.21 (1.1-1.34) | <0.0001 |  |  | 1.14 (1.07-1.22) | <0.0001 |  |  |
| **Cystic fibrosis** |  |  | 1.75 (1.41-2.18) | <0.0001 |  |  |  |  |
| **Interstitial lung disease** |  |  |  |  |  |  | 3.11 (1.76-5.5) | <0.0001 |
| **Cancer** | 1.4 (1.18-1.65) | <0.0001 | 1.39 (1.22-1.58) | <0.0001 | 1.38 (1.24-1.54) | <0.0001 | 1.86 (1.53-2.26) | <0.0001 |
| **Respiratory disease** | 1.26 (1.1-1.44) | <0.0001 |  |  |  |  | 1.38 (1.2-1.6) | <0.0001 |
| **Ischemic heart disease** | 1.37 (1.15-1.62) | <0.0001 | 1.33 (1.17-1.52) | <0.0001 | 1.41 (1.26-1.57) | <0.0001 | 1.4 (1.13-1.72) | <0.0001 |
| **Heart failure** | 1.27 (1.08-1.5) | 1e-04 | 1.55 (1.37-1.76) | <0.0001 | 1.69 (1.51-1.89) | <0.0001 | 1.76 (1.44-2.16) | <0.0001 |
| *Basic treatments* |  |  |  |  |  |  |  |  |
| **System inhibitors** | 1.16 (1.02-1.33) | 0.0026 |  |  |  |  |  |  |
| **NSAIDs** | 1.29 (1.14-1.46) | <0.0001 | 1.22 (1.11-1.34) | <0.0001 | 1.18 (1.1-1.27) | <0.0001 |  |  |
| **Direct oral anticoagulants** |  |  |  |  |  |  | 1.34 (1.12-1.6) | <0.0001 |
| **Heparin** |  |  |  |  | 1.67 (1.43-1.95) | <0.0001 | 3.86 (2.82-5.27) | <0.0001 |
| **Broncodilators** | 1.44 (1.23-1.7) | <0.0001 | 1.43 (1.29-1.59) | <0.0001 | 1.44 (1.32-1.57) | <0.0001 |  |  |
| **Immunosuppressants** | 2.15 (1.5-3.08) | <0.0001 |  |  | 1.62 (1.31-2.01) | <0.0001 |  |  |
| **Chronic systemic steroids** | 1.3 (1.06-1.59) | 0.001 | 1.87 (1.58-2.22) | <0.0001 | 2.21 (1.92-2.55) | <0.0001 | 2.9 (2.33-3.62) | <0.0001 |
| **AUC** | 0.7732 (0.7638-0.7826) | | 0.8143 (0.8072-0.8214) | | 0.8263 (0.8211-0.8316) | | 0.8685 (0.8576-0.8794) | |

First period from March 1 to June 30, 2020. Second period from July 1st until December 28, 2020. Third period from December 29, 2020 to December 13, 2021. Fourth period (Omicron) from December 14, 2021 to January 9, 2022.

COVID-19 medication: remdesivir, nirmatrelvir/ritonavir, sotrovimab or dexamethasone.

Online Figure 1. Description of the main comorbidities related to death by COVID-19 by periods of the pandemic.

It shows all possible combinations of these comorbidities plotted as a function of the number of deaths (vertical axis) and percentage of death (horizontal axis) of individuals with that specific combination who die in each period and colored as a function of the total number of individuals in each combination. Those combinations that exceed either 75% mortality (in the case of 100% with a minimum of 3 individuals that died) or 25 individuals dying have been identified in the graph. X axis limited to 60 deaths. Comorbidities above that number are indicated in the 60 line with the number of deaths associated.

Online Figure 2. Description of the main comorbidities combinations related to death by COVID-19 by periods of the pandemic.

It shows, for each comorbidity, among all the individuals that died with that specific comorbidity, the percentage of deaths happening when combined with different number of comorbidities . Moreover, the percentage of death printed in each column is the maximum death percentage each comorbidity has in the different number of possible combinations located in that combination number specifically. Additionally, the black line shows the number of patients (right vertical axis) that died who had each comorbidity, by itself or in combination with other comorbidities

Online Figure 3. Description of the main comorbidities related to adverse evolution by periods of the pandemic.

It shows all possible combinations of these comorbidities plotted as a function of the number of individuals with adverse evolution (vertical axis) and the percentage of adverse evolution (horizontal axis) of individuals with that specific combination who have adverse evolution in each period and colored as a function of the total number of individuals in each combination. Those combinations that exceed either 75% of adverse evolution (in the case of 100% with a minimum of 3 individuals with adverse evolution) or 25 individuals with adverse evolution have been identified in the graph. X axis limited to 100 adverse events. Comorbidities above that number are indicated in the 100 line with the number of adverse events associated.

Online Figure 4. Description of the main comorbidities combinations related to adverse evolution by periods of the pandemic.

It shows, for each comorbidity, among all the individuals that had adverse evolution with that specific comorbidity, the percentage of adverse evolution happening when combined with different number of comorbidities. Moreover, the percentage of adverse evolution printed in each column is the maximum adverse evolution percentage each comorbidity has in the different number of possible combinations located in that combination number specifically. Additionally, the black line shows the number of patients (right vertical axis) that had adverse evolution who had each comorbidity, by itself or in combination with other comorbidities.

Online Figure 5. Description of the main comorbidities related to hospital admissions by periods of the pandemic.

It shows all possible combinations of these comorbidities plotted as a function of the number of individuals with hospital admission (vertical axis) and the percentage of hospital admission (horizontal axis) of individuals with that specific combination who had hospital admission in each period and colored as a function of the total number of individuals in each combination. Those combinations that exceed either 75% of hospital admission (in the case of 100% a minimum of 6 individuals that were admitted to hospital) or 100 individuals with hospital admission have been identified in the graph. X axis limited to 300 hospital admissions. Comorbidities above that number are indicated in the 300 line with the number of hospital admissions associated.

Online Figure 6. Description of the main comorbidities combinations related to hospital admission by periods of the pandemic.

It shows, for each comorbidity, among all the individuals that had hospital admission with that specific comorbidity, the percentage of hospital admission happening when combined with different number of comorbidities. Moreover, the percentage of hospital admission printed in each column is the maximum hospital admission percentage each comorbidity has in the different number of possible combinations located in that combination number specifically. Additionally, the black line shows the number of patients (right vertical axis) that had hospital admission who had each comorbidity, by itself or in combination with other comorbidities.
